# Physical activity and central adiposity in a cohort of African-American adults

**DOI:** 10.1186/s40608-017-0170-4

**Published:** 2017-11-07

**Authors:** Sean McGrath, Danielle Brazel, Lara Dugas, Guichan Cao, Ramon Durazo-Arvizu, Amy Luke

**Affiliations:** Department of Public Health Sciences, Stritch School of Medicine, Loyola University Chicago, 2160 S. First Ave, Maywood, IL 60153 USA

**Keywords:** Visceral adipose tissue, Waist circumference, Moderate-to-vigorous physical activity, Sedentary behavior

## Abstract

**Background:**

Visceral adipose tissue (VAT) is known as an independent predictor of cardiometabolic risk and all-cause mortality, while increased physical activity has been shown to improve cardiometabolic risk. The purpose of the present study was to determine whether or not there is an association between objectively-measured physical activity and VAT in a community-based cohort of African-American adults, a population at higher-than-average risk for cardiometabolic diseases.

**Methods:**

Free-living physical activity was quantified using accelerometry while VAT and abdominal subcutaneous fat were estimated using dual x-ray absorptiometry in a cohort of African Americans, ages 30–50 years, enrolled in the Modeling the Epidemiologic Transition Study. Univariate and multivariate analyses were used to determine the degree of association comparing moderate-to-vigorous physical activity (MVPA), vigorous activity, and sedentary behavior with measures of central adiposity including VAT, subcutaneous fat, and waist circumference.

**Results:**

A total of 271 individuals with complete data were included in the analyses. Women, on average, had significantly more VAT and abdominal subcutaneous fat than men. There were statistically significant inverse univariate correlations between MVPA and measures of abdominal adiposity (−0.30, *p* < 0.001) and activity counts and adiposity (−0.31, *p* < 0.001) among both sexes. These significant associations remained after controlling for age, sex, and smoking status; neither alcohol consumption nor employment status were associated with abdominal adiposity. Time in sedentary behavior was not meaningfully associated with central adiposity in either sex (women: −0.02, *p* = 0.79; men: −0.21, *p* = 0.04).

**Conclusions:**

To our knowledge, this study is the first to identify significant inverse associations between MVPA and measures of central adiposity among African American adults from a community-based cohort using an objective measure of physical activity and a validated quantitative imaging technique.

## Background

Excess accumulation of visceral adipose tissue (VAT) has been shown to be an independent predictor of insulin resistance, type 2 diabetes, dyslipidemia, and non-alcoholic fatty liver disease [1–9], all of which are associated with increased cardiometabolic risk and are predictors of all-cause mortality [10, 11]. VAT is the most pathogenic fat deposit type and has been found to play a central role in obesity [8]. Recently, it was determined that VAT is a stronger predictor of morbidity and mortality than body weight change [9]. This may be due, at least in part, to the metabolic activity of VAT, which increases release of free fatty acids and pro-inflammatory cytokines relative to subcutaneous fat [12–15].

Physical activity has been shown to improve cardiometabolic risk. Laboratory testing shows higher intensity and higher frequency of exercise bouts and training are associated with increased insulin sensitivity, glucose tolerance, and fasting triglycerides [16–19]. Multiple professional and medical organizations provide physical activity guidelines to promote optimal health including the American College of Sports Medicine (ACSM), the American Heart Association (AHA), and the U.S. Department of Health and Human Services. The ACSM and AHA guidelines recommend that U.S. adults partake in moderate-intensity aerobic physical activity for at least 30 min on five or more days per week or vigorous-intensity aerobic activity for 20 min on at least 3 days throughout the week [20]. Federal guidelines are similar, differing only in the recommendation of 75 min of vigorous aerobic activity over the course of a week [21]. In contrast, sedentary behavior has been associated with increased cardiometabolic risk [22]. In the U.S. it has been repeatedly observed that most adults do not meet the recommendations for physical activity and instead spend a majority of their time either in sedentary or light-intensity activity [23].

Understanding the mechanisms through which physical activity potentially impacts cardiometabolic risk would help inform targeted interventions. Describing the relationship between VAT and physical activity in free-living populations is a first step in this understanding. To date, much of the research in this area has focused on controlled laboratory experiments or, when conducted at the community- or population-level, has utilized self-reported physical activity and a proxy for VAT and waist circumference. Two recent reports have described inverse associations between objectively measured physical activity and VAT in large samples, though both examined predominantly white participant groups i.e., ADDITION-PRO, a study among Danish individuals at high risk for type 2 diabetes recruited from primary care [24] and the Framingham Third Generation and Omni II cohorts [25]. Little is known, however, about the relationship between physical activity and central adiposity or VAT among African-Americans adults. To gain further insight into the association between physical activity and VAT in this population at high risk of cardiometabolic disease [26], we describe the cross-sectional association between physical activity measured using accelerometry and VAT measured using dual x-ray absorptiometry (DXA) in a community-based sample of African-American adults participating in the Modeling the Epidemiologic Transition Study (METS) [27].

## Methods

### Study population and institutional review board

METS is a multi-country, longitudinal cohort study designed to examine the relationship between objectively-measured physical activity and weight gain among five community-based samples of adults, predominantly of African descent. Five hundred adults, aged 25 to 45 years, were enrolled between January 2010 and December 2011 within communities in each of the five countries: Ghana, South Africa, Jamaica, Seychelles, and the United States. The study site in the United States is Maywood, IL, a near-western suburb of Chicago in which the majority of residents are African-American. A detailed description of all methods, including recruitment methods in each site, has been previously published [27]. In brief, all city blocks in Maywood were randomized and door-to-door canvassing was conducted until enrollment was complete. Exclusion criteria, as detailed previously, included conditions that would prevent participation in physical activity, such as lower extremity disability and degenerative joint diseases, as well as current pregnancy [27]. The protocol for METS was approved by the Institutional Review Board of Loyola University Chicago and written informed consent was obtained from all participants.

The participants in METS have been followed annually since 2011 for measurement of weight, at a minimum. During the third annual follow-up examination, as a component of the ancillary study, Vitamin D Ancillary, a measurement of body composition using DXA was added to the protocol; anthropometrics and physical activity by accelerometry were measured and change in health status was obtained. An addendum to the original informed consent was signed by all participants covering this additional measurement of body composition. The data presented in this report were all obtained during the third annual follow-up examination for METS.

### Measurements

Physical activity was measured objectively using the Actical accelerometer (Respironics/Mini-Mitter, Bend, OR). Participants were asked to wear the accelerometer behind the right hip on a velcro belt and keep it on 24-h per day over the subsequent 8 days with the exceptions of bathing, showering, and swimming. Activity conducted between the hours of 7 am and 11 pm daily were used in these analyses. As reported previously [28], raw data were downloaded from the accelerometers and assessed via a SAS macro program inferring non-wear time from 90 or more minutes of continuous zero activity counts. A valid day of physical activity monitoring was defined as one having ≥10 h of wear time. Only those participant files with four or more valid days of physical activity monitoring were included in these analyses.

Sedentary, moderate, and vigorous activity levels were defined using published criteria: sedentary <100 counts per minute (cpm), moderate activity 1535–3959 cpm and vigorous activity ≥3960 cpm [29, 30]. Minutes defined as comprising sedentary, moderate, vigorous, or moderate plus vigorous activity (MVPA) are presented as the total minutes per day accumulated in 1-min intervals. Data are also presented as activity counts per minute as an overall measure of average physical activity intensity. Participants were classified as meeting physical activity guidelines if they accumulated at least 30 min of MVPA per day on average.

Body composition was measured using DXA (Hologic Discovery W configured with software version 12.1, Bedford, MA). Visceral adipose tissue volume and area were calculated using algorithms developed specifically for the Hologic Discovery W DXA instrument [31, 32]. Total abdominal fat in all participants was measured at a site just superior to the iliac crest, as determined from whole body scans, to minimize interference from bone pixels. Traditional DXA estimates of abdominal fat include both subcutaneous and VAT, however, it is possible to differentiate subcutaneous fat in the lateral aspects of the abdomen from muscle or lean tissue [31, 32]. Quantification of lateral subcutaneous fat allowed approximation of anterior and posterior subcutaneous fat, yielding an estimation of total abdominal subcutaneous fat, ie, lateral plus anterior and posterior volumes. Total abdominal subcutaneous fat was then subtracted from total abdominal fat to yield VAT. VAT and subcutaneous fat are expressed as volume (cm^3^). Additionally, VAT area was calculated and used to determine whether or not participants had excess visceral adiposity, defined as >100 cm^2^ [33].

Visceral adipose tissue has historically been difficult to measure. Many methods have been used including MRI, computed tomography, ultrasonography, and anthropometric estimations such as waist circumference. These methods have significant drawbacks including cost, radiation exposure, and reproducibility. Dual x-ray absorptiometry measurements of VAT have been shown to be as accurate as computed tomography with less radiation exposure [34] and less expensive than MRI [35].

Weight (kg) and height (m) measurements were made on all participants while wearing light clothing and no shoes. Weight was measured to the nearest 0.1 kg using a standard calibrated balance (Seca 770, Hamburg, Germany). Height was measured to the nearest 0.1 cm using a stadiometer (e.g. Invicta Stadiometer, Invicta, London, UK) with the participant’s head held in the Frankfort plane. Waist circumference was measured to the nearest 0.1 cm at the umbilicus and hip at the point of maximum extension of the buttocks. Body mass index (BMI) was calculated as kg/m^2^.

A positive smoking status was defined as currently smoking at least one cigarette per day, while positive alcohol consumption was defined as consuming any alcohol during a typical week. Participants were coded as employed if they had worked for pay in the previous month.

### Statistical analysis

All analyses were conducted in Stata Version 12 (College Station, TX, USA). Statistical significance was accepted at *p* < 0.05. Descriptive statistics including mean levels and distributions were used to summarize participant characteristics. Prevalences of health status indicators, i.e. obesity and excess visceral adiposity, were calculated for each sex. In addition, descriptive characteristics of the physical activity variables were calculated and included only those individuals with valid data as determined by the inclusion criteria previously described. Univariate analyses were conducted to determine partial correlation coefficients between parameters of physical activity and central adiposity by sex. Multivariate analyses were conducted using linear regression to describe the relationship between physical activity and central adiposity controlling for age, sex, and smoking status in the combined model and for age and smoking status in the sex-specific models.

## Results

### Participant characteristics

A total of 297 participants were examined during the third annual follow-up investigation for METS in Maywood (60% retention from baseline), of which there were complete DXA and physical activity data on 271 participants available for analysis. There were no differences in baseline weight, height, waist circumference or BMI among those participants who completed the third follow-up examination and those lost to follow-up (Table 1; all *p* > 0.2). Among women, baseline physical activity parameters, including minutes of MVPA, did not differ between completers of the third examination and those lost to follow-up. Among men, however, baseline age was statistically significantly older (36.7 ± 6.3 vs 34.7 ± 6.1 y, respectively; *p* < 0.05) and moderate-to-vigorous physical activity was lower among those who remained in METS compared to those lost to follow-up (38.0 ± 3.9 vs 28.5 ± 2.5 min/day; p < 0.05). These differences could not be explained by the employment status or employment type of the completers versus those lost to follow-up.

**Table 1:** Baseline characteristics of participants at the third follow-up examination and those lost to follow-up (mean ± SD)

	Women		Men	
	Third Follow-up Examination (*n* = 161)	Lost to Follow-up (*n* = 96)	Third Follow-up Examination (*n* = 110)	Lost to Follow-up (*n* = 135)
Age (y)	35.0 ± 6.3	35.0 ± 6.2	36.7 ± 6.3*	34.7 ± 6.1*
Weight (kg)	92.1 ± 25.5	90.9 ± 22.8	92.8 ± 27.4	92.8 ± 22.5
Height (cm)	163.8 ± 6.3	164.3 ± 6.0	175.9 ± 6.5	177.2 ± 6.6
Body Mass Index (kg/m^2^)	34.3 ± 9.2	33.7 ± 8.3	29.9 ± 8.2	29.6 ± 6.9
Waist Circumference (cm)	102.6 ± 20.7	100.5 ± 17.8	98.4 ± 22.9	96.2 ± 20.2
MVPA (min/d)	14.5 ± 17.2	16.6 ± 18.9	28.8 ± 27.0	37.6 ± 41.3
Vigorous PA (min/d)	1.4 ± 3.4	1.2 ± 3.1	2.7 ± 4.6*	6.3 ± 14.1*

*p < 0.05

Characteristics of those participants who completed the third annual follow-up examination, for whom there were complete body composition and physical activity data, are presented in Table 2. The study population is composed of middle-aged African Americans with an average BMI of 35.0 kg/m^2^ for women and 30.2 kg/m^2^ for men. The cohort had very high prevalences of whole-body obesity, as defined by BMI ≥30 kg/m^2^, and of excess visceral adiposity, especially among the women. The participants reported relatively high proportions of current smokers and consumers of alcohol, particularly among the men. A large majority of both women and men were employed at the time of the third follow-up examination.

**Table 2:** Participant characteristics at third follow-up examination (mean ± SD; %)

	Women (*n* = 161)	Men (*n* = 110)	Total (*n* = 271)
Age (y)	38.1 ± 6.4	39.7 ± 6.6	38.8 ± 6.5
Weight (kg)	91.0 ± 24.1	89.6 ± 26.1	90.4 ± 24.9
Body mass index (kg/m^2^)	35.0 ± 9.2	30.2 ± 8.3	33.0 ± 9.1
Waist circumference (cm)	104.8 ± 19.5	100.1 ± 20.8	102.9 ± 20.1
Hip circumference (cm)	119.7 ± 16.5	110.5 ± 15.0	115.9 ± 16.5
Whole body lean mass (kg)	55.0 ± 10.4	67.8 ± 12.1	60.2 ± 12.8
Whole body fat mass(kg)	36.0 ± 15.3	21.8 ± 15.7	30.2 ± 16.9
Body fat (%)	38.4 ± 7.9	22.2 ± 9.5	31.8 ± 11.7
Visceral adipose tissue (cm^3^)	686 ± 366	562 ± 362	636 ± 369
Abdominal subcutaneous tissue (cm^3^)	2504 ± 902	1323 ± 1039	2025 ± 1120
% Obese	68.8	40.3	57.3
% Excess Visceral Adiposity^a^	64.8	45.4	57.0
Activity counts (ct/min)	128.2 ± 69.2	185.0 ± 115.1	151.3 ± 94.7
Moderate-to-vigorous intensity activity (min/d)^b^	13.0 ± 13.6	29.8 ± 30.2	19.8 ± 23.3
Vigorous intensity activity (min/d) ^b^	1.0 ± 2.5	2.9 ± 5.9	1.8 ± 4.3
Sedentary behavior (min/d)^b^	214.4 ± 46.0	209.0 ± 52.7	212 ± 49
% Meeting PA Guidelines^c^	15.3	36.1	23.7
% Current Smoker^d^	27.5	60.9	41.1
% Alcohol Consumption^e^	36.9	61.8	47.0
% Employed^f^	81.3	82.6	81.8

^a^Defined as >100 cm^2^ VAT

^b^As measured in 1-min bouts

^c^Defined as >30 min/d of MVPA

^d^Defined as currently smoking at least 1 cigarette per day

^e^Defined as consuming any alcoholic beverages during a typical week

^f^Defined as working for pay in the previous month

### Body composition by DXA

As presented in Table 2, participants had an average of 634 cm^3^ of visceral adipose tissue and 2007 cm^3^ of abdominal subcutaneous adipose tissue. Men had less subcutaneous adipose and visceral adipose tissue than women, with men having 1323 cm^3^ of SAT and 562 cm^3^ of VAT compared to women who had 2504 cm^3^ of SAT and 686 cm^3^ of VAT.

### Physical activity by accelerometer

On average, participants undertook 19.6 min per day of MVPA, 1.8 min of vigorous intensity activity, and 213.3 min of sedentary behavior. Men tended to participate in twice the amount of MVPA and vigorous intensity activity as women. On average men partook in 29.8 min per day of MVPA and 2.9 min per day of vigorous activity as compared to women who averaged 13.0 min per day of MVPA and 1.0 min per day of vigorous activity. Among men, 36.1% met the U.S. physical activity guidelines of 30 min/day of MVPA while only 15.3% of the women met the same guidelines (*p* < 0.001).

### Relationship between visceral adipose tissue and physical activity

Table 3 presents the univariate correlation coefficients between measures of physical activity and measures of central adiposity. There were statistically significant negative associations between MVPA and VAT, abdominal subcutaneous adipose tissue, and waist circumference in both sexes. Likewise, activity counts were consistently significantly associated with measures of central adiposity in both women and men. In contrast, correlations between vigorous intensity activity or sedentary behavior and central adiposity differed between the sexes. Among women, vigorous activity was significantly, inversely associated with the measures of adiposity while sedentary behavior was not significantly associated at all. In men, no statistically significant association was found between vigorous activity and central adiposity while sedentary behavior was found to be inversely associated. It must be noted, however, that the association between sedentary behavior and adiposity was opposite from what would be expected, i.e., an inverse association was observed rather than the anticipated positive association.

**Table 3:** Univariate associations between markers of central adiposity and physical activity parameters by sex (correlation coefficient [*p*-value])

	VAT (cm^3^)	SAT (cm^3^)	Waist circumference (cm)
Total (*n* = 271)			
Activity counts (ct/min)	−0.31 [0.001]	−0.34 [0.001]	−0.23 [0.001]
MVPA (min/d)^a^	−0.30 [0.001]	−0.37 [0.001]	−0.24 [0.001]
Vigorous intensity activity (min/d)^a^	−0.21 [0.001]	−0.25 [0.001]	−0.19 [0.002]
Sedentary behavior (min/d)^a^	−0.09 [0.13]	−0.09 [0.13]	−0.07 [0.25]
Women (*n* = 161)			
Activity counts (ct/min)	−0.24 [0.002]	−0.16 [0 .04]	−0.15 [0.05]
MVPA (min/d)^a^	−0.25 [0.001]	−0.21 [0.008]	−0.19 [0.02]
Vigorous intensity activity (min/d)^a^	−0.24 [0.003]	−0.21 [0.008]	−0.20 [0.01]
Sedentary behavior (min/d)^a^	−0.02 [0.79]	−0.06 [0.49]	−0.03 [0.71]
Men (*n* = 110)			
Activity counts (ct/min)	−0.32 [0.001]	−0.28 [0.003]	−0.27 [0.005]
MVPA (min/d)^a^	−0.29 [0.002]	−0.26 [0.006]	−0.24 [0.01]
Vigorous intensity activity (min/d)^a^	−0.15 [0.11]	−0.16 [0.09]	−0.18 [0.06]
Sedentary behavior (min/d)^a^	−0.21 [0.03]	−0.20 [0.03]	−0.19 [0.04]

^a^As measured in 1-min bouts

Results of the multivariate regression analyses are presented in Table 4, for the total cohort as well as for women and men, separately. In the total cohort, after controlling for age, sex, and smoking status, statistically significant inverse associations were observed between all measures of physical activity and the outcome variables of VAT, SAT and waist circumference (all *p* < 0.01). When stratified by sex and after controlling for age and smoking status, slight differences in the associations between physical activity parameters and measures of central adiposity were observed between the women and the men. Among women, statistically significant associations were observed for all measures of activity and of adiposity; smoking status was not independently associated with VAT, SAT or waist circumference. Among men, however, the associations between activity counts and MVPA and measures of central adiposity remained statistically significant, whereas vigorous activity was only marginally associated. In contrast to women, smoking status was independently associated with VAT and SAT among men. Only a relatively small proportion of the variance in VAT, SAT or waist circumference could be explained by the covariates used in the sex-stratified models. Over 30% of the variance in SAT could be explained by the model which included both sexes, however, this is due to the significant difference in SAT volume between women and men.

**Table 4:** Regression coefficients and intercepts for the relationship between parameters of physical activity and central adiposity, adjusting for age and smoking status, total cohort & by sex (*p*-value)

	Intercept	β_1_ Activity Parameter	β_2_ Age	β_3_ Sex	β_4_ Smoking Status	SEE	R^2^
Total (*n* = 271)							
VAT (cm^3^)							
Activity Counts (ct/min)	484 (0.001)	−1.1 (0.001)	7.9 (0.02)	56.0 (0.25)	−69.7 (0.14)	348	0.13
MVPA (min/d)	434 (0.003)	−4.0 (0.001)	7.1 (0.03)	49.4 (0.32)	−65.2 (0.17)	351	0.11
Vigorous PA (min/d)	311 (0.03)	−15.5 (0.003)	8.7 (0.01)	83.4 (0.09)	−84.4 (0.08)	355	0.09
SAT (cm^3^)							
Activity Counts (ct/min)	1701 (0.001)	−2.2 (0.001)	5.8 (0.51)	956 (0.001)	−325 (0.009)	932	0.32
MVPA (min/d)	1630 (0.001)	−9.1 (0.001)	3.9 (0.66)	928 (0.001)	−311 (0.01)	932	0.32
Vigorous PA (min/d)	1351 (0.001)	−37.6 (0.007)	7.5 (0.39)	1003 (0.001)	−355 (0.005)	940	0.31
Waist Circumference (cm)							
Activity Counts (ct/min)	98.8 (0.001)	−0.05 (0.001)	0.28 (0.13)	1.95 (0.47)	−2.89 (0.27)	19.6	0.07
MVPA (min/d)	97.1 (0.001)	−0.18 (0.001)	0.24 (0.19)	1.51 (0.58)	−2.63 (0.31)	19.7	0.07
Vigorous PA (min/d)	91.8 (0.001)	−0.83 (0.004)	0.32 (0.09)	2.81 (0.29)	−3.48 (0.18)	19.7	0.6
Women (*n* = 161)							
VAT (cm^3^)							
Activity Counts (ct/min)	506 (0.004)	−1.4 (0.001)	9.6 (0.03)	–	−6.6 (0.92)	355	0.09
MVPA (min/d)	429 (0.01)	−7.8 (0.001)	9.4 (0.03)	–	0.23 (0.99)	354	0.09
Vigorous PA (min/d)	431 (0.01)	−39.6 (0.001)	7.9 (0.07)	–	−17.0 (0.79)	356	0.08
SAT (cm^3^)							
Activity Counts (ct/min)	2645 (0.001)	−2.0 (0.07)	4.8 (0.67)	–	−257 (0.12)	898	0.04
MVPA (min/d)	2551 (0.001)	−13.4 (0.02)	5.0 (0.65)	–	−241 (0.14)	890	0.05
Vigorous PA (min/d)	2566 (0.001)	−79.8 (0.009)	2.5 (0.82)	–	−270 (0.09)	888	0.06
Waist Circumference (cm)							
Activity Counts (ct/min)	99.8 (0.001)	−0.05 (0.04)	0.29 (0.23)	–	0.35 (0.92)	19.5	0.03
MVPA (min/d)	97.3 (0.001)	−0.30 (0.01)	0.29 (0.22)	–	0.67 (0.84)	19.4	0.04
Vigorous PA (min/d)	97.5 (0.001)	−1.64 (0.01)	0.23 (0.33)	–	0.03 (0.99)	19.4	0.04
Men (*n* = 110)							
VAT (cm^3^)							
Activity Counts (ct/min)	535 (0.01)	−0.88 (0.003)	7.0 (0.17)	–	−146 (0.03)	339	0.15
MVPA (min/d)	478 (0.03)	−2.9 (0.01)	6.4 (0.22)	–	−141 (0.04)	343	0.13
Vigorous PA (min/d)	295 (0.15)	−10.0 (0.08)	10.0 (0.05)	–	−168 (0.02)	349	0.10
SAT (cm^3^)							
Activity Counts (ct/min)	1717 (0.01)	−2.3 (0.01)	7.1 (0.63)	–	−410 (0.04)	996	0.11
MVPA (min/d)	1582 (0.01)	−7.8 (0.02)	5.4 (0.72)	–	−395 (0.05)	1004	0.10
Vigorous PA (min/d)	1096 (0.07)	−28.3 (0.09)	15.0 (0.31)	–	−467 (0.02)	1016	0.08
Waist Circumference (cm)							
Activity Counts (ct/min)	99.1 (0.001)	−0.04 (0.02)	0.32 (0.28)	–	−6.8 (0.09)	20.0	0.10
MVPA (min/d)	96.4 (0.001)	−0.14 (0.04)	0.29 (0.34)	–	−6.6 (0.10)	20.1	0.09
Vigorous PA (min/d)	87.9 (0.001)	−0.64 (0.05)	0.47 (0.11)	–	−7.9 (0.05)	20.2	0.08

No statistically significant association was observed between sedentary behavior and any measure of central adiposity. Likewise, alcohol consumption and employment status were not included in the multivariate analyses due to a consistent lack of association with outcomes (*p* > 0.5).

## Discussion

These analyses demonstrate significant inverse associations between physical activity parameters and all measures of central adiposity in this community-based sample of African-American adults, more than half of whom were classified as obese based on BMI. The strongest of these univariate associations in both women and men were between MVPA and VAT, while abdominal SAT and waist circumference, indirect measures of central adiposity, were also significantly negatively correlated with MVPA. These univariate associations remained statistically significant after controlling for age, sex, and current smoking status. In contrast to expectations, however, time spent in sedentary behavior was not positively associated with any parameter of central adiposity.

Similar to the current study, Murabito et al. [25] examined the relationship between objectively measured physical activity and VAT among men and women enrolled in the Framingham Third Generation and Omni II cohorts. In concert with our results, these authors observed an inverse association between MVPA and VAT for both men and women with no significant relationship identified for sedentary time and VAT.

Results from other studies have produced mixed results regarding an association between abdominal adiposity and objective measures of physical activity. One study conducted by Smith et al. [36] using computed tomography to quantify VAT and concluded that physical activity, when measured using accelerometry and expressed as MVPA or total active time, was not associated with VAT among men but was among women. In contrast, Philipsen et al. [24] conducted a similar study measuring VAT using ultrasonography and, similar to the present study’s results, reported an inverse association between physical activity and visceral adipose tissue in both sexes. Interestingly, both the Smith et al. and Philipsen et al. studies combined physical activity from all intensity categories into one global physical activity classification. The present analyses were conducted with the physical activity intensity categories kept separate in order to assess whether or not MVPA or vigorous physical activity versus minutes of sedentary behavior impacted central adiposity. Our findings have shown that statistically significant associations exist for MVPA and all parameters of central adiposity, which in this case include VAT, subcutaneous adipose tissue, and waist circumference. The present study found no significant relationship between vigorous activity and adiposity among men. Among women, however, there were significant associations between minutes of vigorous activity and measures of adiposity, despite the fact that women only recorded 1 min of vigorous activity per day on average.

Previously, well-controlled studies have investigated the impact of initiating exercise programs on the reduction of VAT [37, 38] and found a dose-response relationship between physical activity intensity and the reduction in VAT. Individuals who exercise at higher intensities have greater reductions in VAT than those exercising moderately or following dietary restrictions only [37, 38]. One study concluded the greatest reduction in mortality risks when comparing inactive to moderately active groups across all levels of adiposity [39]. Moving from the inactive to the moderately inactive group required a physical activity energy expenditure between 90 and 110 kcal/day, or in other words, about 20 min of brisk walking per day. This indicates that even small increases in activity in otherwise inactive individuals may significantly benefit health on a broad scale. Interestingly, another study showed the combination of aerobic exercise and strength training has a modest reduction of VAT but both separately had a significant VAT decrease [8]. This study found a greater effect in men, possibly due to men having more abdominal fat at baseline and thus making them more susceptible to abdominal adiposity changes. This same study also showed that exercise programs without caloric restriction can reduce VAT. Another recent investigation in overweight and obese individuals found that diet restriction resulted in greater weight loss but exercise produced greater VAT reduction [9]. The authors explain this is likely due to caloric restriction decreasing both muscle and fat mass versus exercise increases muscle mass while decreasing fat mass. This suggests that VAT may be a more useful clinical marker of obesity management and intervention. In our study, the lack of association between vigorous physical activity and VAT among men may reflect the overall low levels of objectively measured vigorous activity we captured in this study. It should, however, be noted that the present study was designed to capture habitual physical activity levels rather than exercise intensity or intervention, and these data indicate low exercise levels.

Our study had several limitations; the first being the cross-sectional nature. It is not possible to infer whether increasing physical activity might decrease VAT. Understanding changes in abdominal adiposity over time in relationship to habitual physical activity levels will provide guidance for clinical applications. Secondly, although statistically significant associations were seen, our participants participated in very little vigorous activity; only 1.8 min per day on average for men and women combined. Having a population that engaged in more vigorous activity may allow for stronger associations. The present population-based study was designed as an observational assessment rather than any type of exercise intervention, therefore, caution should be taken when interpreting these results through the lens of exercise.

Strengths of the present study include the objective measurement of both physical activity and abdominal fat and the community-based sample of study participants. The African-American participants enrolled in the present study mirror populations at increased risk for cardiometabolic diseases including type 2 diabetes. The negative health impact of increased levels of VAT is substantial. Increased volume of VAT has been shown to increase dyslipidemia, insulin resistance, non-alcoholic fatty liver disease and all-cause mortality [1–3, 6, 8, 10], therefore, it is important to identify the level of MVPA and vigorous intensity physical activity required to reduce VAT in this population. This study provides a baseline for further prospective analysis to determine the association between physical activity and visceral adipose tissue and its effect on metabolic risk. Longitudinal analyses are needed to fully understand how visceral adipose tissue changes with physical activity.

## Conclusions

To our knowledge, this study is the first to identify associations between parameters of physical activity and measures of central adiposity among African American adults from a community-based cohort. Major strengths of this study include objective measure of physical activity and a validated quantitative imaging technique. This study is unique in that normal physical activity level, rather an exercise intervention, was assessed and may have important clinical implications for future obesity management.

## Abbreviations

ACSM: American College of Sports Medicine
AHA: American Heart Association
DXA: Dual x-ray absorptiometry
METS: Modeling the epidemiologic transition study
MRI: Magnetic resonance imaging
MVPA: Moderate-to-vigorous physical activity
SAS: Statistical analysis system
SAT: Subcutaneous adipose tissue
VAT: Visceral adipose tissue
WDXA: W configured dual x-ray absorptiometry
